# Enhancing the annual yield via nitrogen fertilizer application optimization in the direct seeding ratoon rice system

**DOI:** 10.3389/fpls.2024.1366718

**Published:** 2024-03-13

**Authors:** Yang Li, Zuolin Zhang, Benfu Wang, Zhisheng Zhang, Yiyue Lin, Jianping Cheng

**Affiliations:** ^1^ Hubei Key Laboratory of Food Crop Germplasm and Genetic Improvement & Key Laboratory of Ministry of Agriculture and Rural Affairs for Crop Molecular Breeding, Food Crops Institute, Hubei Academy of Agricultural Sciences, Wuhan, China; ^2^ College of Agriculture, Yangtze University, Jingzhou, China

**Keywords:** direct seeding ratoon rice, nitrogen fertilizer, dry matter mass accumulation, leaf senescence rate, root activity

## Abstract

Direct seeding ratoon rice (DSRR) system is a planting method that can significantly increase grain yield, improving light and temperature utilization efficiency and reducing labor input. However, the current nitrogen fertilizer management method which does not aim at the seedling emergence and development characteristics of DSRR just is only based on the traditional method of transplanting ratoon rice, and which is not conducive to the population development and yield improvement. To determine the suitable nitrogen fertilizer application optimization, we set four nitrogen fertilizer application treatments (N0, no nitrogen fertilizer; N1, traditional nitrogen fertilizer; N2, transferring 20% of total nitrogen from basal fertilizer to tillering stage; N3, reducing total nitrogen by 10% from N2 tillering fertilizer) on a hybrid rice “Fengliangyouxiang1 (FLYX1)” and an inbred rice “Huanghuazhan (HHZ)” under DSRR. The effects of treatments on dry matter accumulation, root growth and vigor, leaf area index, leaf senescence rate and yield were investigated. Our results demonstrated that the yield of main crop in N2 treatment was the highest, which was 63.3%, 6.6% and 8.8% higher than that of N0, N1 and N3 treatment, respectively, mainly due to the difference of effective panicle and spikelets number per m^2^. The average of two years and varieties, the annual yield of N2 was significant higher than that of N1 and N3 by 4.94% and 8.55%, respectively. However, there was no significant difference between the annual yields of N1 and N3. N2 treatment had significant effects on the accumulation of aboveground dry matter mass which was no significant difference in 20 days after sowing(DAS), but significant difference in 50 DAS. Meanwhile, the root activity and the leaf senescence rate of N2 treatment was significant lower than that of other treatments. In summary, “20% of total nitrogen was transferred from basal fertilizer to tillering stage” can improve the annual yield and main crop development of DSRR system. Further reducing the use of nitrogen fertilizer may significantly improve the production efficiency of nitrogen fertilizer and improve the planting income in DSRR system.

## 1 Introduction

Ratoon rice (RR) is a planting system in which the dormant buds on the rice stubble survive after harvesting in the main crop, and reasonable cultivation measures are taken to promote the germination and growth of dormant buds into ratoon buds, then heading, filling and fruiting, and harvesting of rice for ratoon crop. In recent years, due to the advantages of high yield, excellent rice quality, and saving labor, fertilizer, seeds, water, pesticides and seedbeds, the planting area has gradually expanded (Peng, 2014; Wang et al., 2023). It is also considered one of the sustainable and promising production systems to increase the frequency of harvesting, and the total area of RR in China has exceeded 1 million hectares (Yu et al., 2022). By adopting reasonable varieties and management measures, the yield of ratoon rice in the first and second seasons can reach 9-10 t ha^-1^ and 5-6 t ha^-1^, respectively. Compared with mid-season rice, RR significantly reduced production resource input, but increased rice farmers’ income, and RR’s environmental footprint was significant lower than that of mid-season rice (Yuan et al., 2019; Peng et al., 2023). Moreover, RR has lower input of agricultural production factors and higher resource utilization efficiency than double-cropping rice (Feng et al., 2013). The planting pattern of RR has made an important contribution to food security in China.

With the increasing of production cost, the further development of light and simplified system is an important measure to reduce production input and increase planting income. The area of direct-seeded rice has been expanding rapidly in recent years because it reduces water consumption, labor requirements, nursery supplies and increases system productivity and resource use efficiency (Jiang et al., 2016; Sha et al., 2019). Direct seeding ratoon rice (DSRR) was formed by combining seeding rice technology and RR technology, which could save cost and improve production efficiency. Some studies have shown that the annual rate of direct regrowing rice can reach 15.7 t ha^-1^, and the yield of ratoon season can exceed 5.5 t ha^-1^ under experimental conditions (Dong et al., 2017). Chen et al. (2018) has demonstrated that the yield of hybrid rice cultivars was significant higher than that of inbred rice cultivars which is due to higher ratoon rate under DSRR. And in the system, it can not only improve grain quality but also mitigate yield-scaled CH_4_ gas emission and total organic carbon loss rate of rice fields (Zhang et al., 2022). But in the meanwhile, lodging is also a potential disadvantage for the development of direct seeded rice (Sinniah et al., 2012). Reasonable population construction is an important way to ensure stable production of DSRR. Fertilizer management, that is, fertilization method and fertilization ratio, is often an important means to determine whether the population is reasonable and the plant productivity is high.

Studies have shown that deep application of base fertilizer 70% as base fertilizer and 30% as topdressing can significantly increase nitrogen content and population dry matter accumulation in soil (Wang et al., 2019). One-time deep fertilization can also significantly improve grain yield, nitrogen use efficiency and reduce greenhouse gas emissions (Li et al., 2021). Delaying the first fertilization time of direct seeding rice can significantly increase yield, delaying leaf senescence and improving population quality (Liu et al., 2019; Li et al., 2023). Real-time and on-the-spot nitrogen management measures can improve nitrogen use efficiency, growth state and yield composition (Ali et al., 2015; Mohanty et al., 2021). However, the above is basically the direct seeding nitrogen management technology of middle rice. Although it is consistent with the direct seeding technology of DSRR, the two systems have a long difference in seeding time, resulting in a big difference in temperature and light resources during direct seeding, and there should be a relatively big difference in nitrogen management technology. At present, most of the fertilization methods under DSRR system refer to the traditional direct-seeding nitrogen fertilizer management technology of middle rice or the traditional transplanting and RR nitrogen fertilizer management technology. In the middle and lower reaches of the Yangtze River, DSRR seeding time is generally in early April, when the temperature has not stabilized above 15°C, and the plant production process such as emergence and tillering is relatively slow (Jiang et al., 2008; Wang et al., 2013). This means that the early stage of rice growth, a period of time after sowing, does not require much external nutrient supply during the rice emergence or seedling stage. Traditional transplanting and replanting rice focuses on heavy application of bottom fertilizer to promote tillering (Yang et al., 2021), or to resist low temperature and cold damage (Zhou et al., 2018). For the characteristics of slow growth in the early stage of direct seeding rice, excessive nutrient supply may lead to loss due to surface runoff on the one hand (Zhang et al., 2018), and to growth of a large number of ineffective tillers on the other hand (Huang et al., 2013), to cause the unhealthy main crop population. However, there are very few studies on nitrogen fertilizer management in the main crop of DSRR.

In view of this, the rational management of nitrogen fertilizer in the main crop under DSRR system is of great significance to whether this system can produce high yield and high efficiency. In order to solve this problem, we conducted a 2-year field experiment under the DSRR system aiming to examine whether the traditional nitrogen application method is suitable for the DSRR and estimate the effect of changes in nitrogen method on the population growth of the main crop, and then explore a more reasonable nitrogen fertilizer management method.

## 2 Materials and methods

### 2.1 Site description

**Figure 1:**
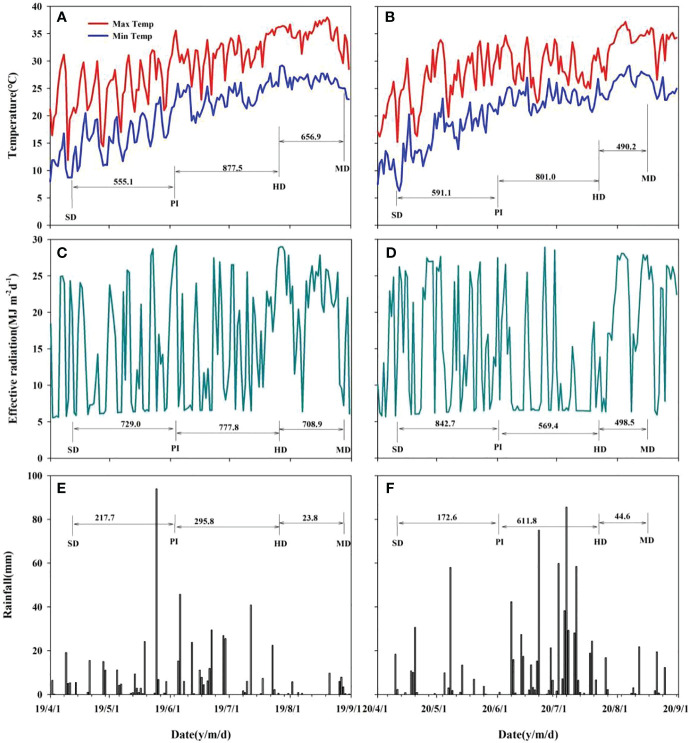
Effective accumulative temperature, effective radiation and rainfall during the main crop growth stage in 2019 **(A, C, E)** and 2020 **(B, D, F)**. SD, sowing date; PI, panicle initiation; HD, heading date; MD, mature date.

Field experiments were conducted at the Sanhu Farm, Jiangling County, Hubei Province, China, during the rice growing seasons of 2019 and 2020. Total N, Olsen phosphorus (P), available potassium (K), organic matter, and pH of the topsoil (0-20 cm layer) were 2.0 g kg^-1^, 15.5 mg kg^-1^, 160 mg kg^-1^, 2.7% and 5.5, respectively. The climate data regarding daily solar radiation, rainfall, and air temperature were collected from a weather station (U30-NRC; Onset Inc., USA) near the experimental field (**Figure 1**).

### 2.2 Experimental design

The experiments were randomized in a complete block design with three replications. The plot size was 100 m^2^ (20 m × 5 m). All plots were ploughed and puddled by rotary cultivator before seed sowing. Two rice cultivars, an inbred Huanghuazhan (HHZ) and a hybrid Fengliangyouxiang1 (FLYX1), were used for the experiment. Both cultivars have been widely grown for ratoon rice in the study region. Four nitrogen management treatments for main crop included N0, N1, N2, and N3. In N0 treatment, no N fertilizer was applied as a zero-N control. In N1 treatment, 180 kg N ha^-1^ was split-applied with 50% as basal nitrogen (BSN) at 1 day before sowing, 20% at tiller nitrogen (TLN) at 20 day after sowing (DAS), and 30% at panicle initiation nitrogen (PIN) at 52 DAS. Total N rate was also 180 kg ha^-1^ for N2 treatment, but it was split-applied with 30% as BSN, 40% as TLN, and 30% as PIN. In N3 treatment, 162 kg N ha^-1^ was applied in three equal splits as BSN, TLN, and PIN, respectively. Detailed information about N treatments was listed in **Table 1**. In all plots, 90 kg P_2_O_5_ ha^-1^ and 75 kg K_2_O ha^-1^ were applied at 1 d before seeding, and 75 kg K_2_O ha^-1^ was applied at PI. The sources of N, P, and K were urea, calcium superphosphate, and potassium chloride, respectively. To minimize leakage between plots, all buds were covered with plastic film installed into a depth of 20 cm below soil surface.

**Table 1:** The rate of N fertilizer application under different treatments.

Treatment	BSN kg ha^-1^	TLN kg ha^-1^	PIN kg ha^-1^	Total N kg ha^-1^
N0	0	0	0	0
N1	90	36	54	180
N2	54	72	54	180
N3	54	54	54	162

BSN, basal nitrogen; TLN, tiller nitrogen; PIN, panicle initiation nitrogen; Total N, total nitrogen application. N0, no N fertilizer; N1, 180 kg N ha^-1^ was split-applied with 50% as basal nitrogen (BSN) at 1 day before sowing, 20% as tiller nitrogen (TLN) at 20 DAS, and 30% as panicle initiation nitrogen (PIN) at 52 DAS. N2, 180 kg N ha^-1^ was split-applied with 30% as BSN at 1 day before sowing, 40% as TLN at 20 DAS, and 30% as PIN at 52 DAS; N3, 162 kg N ha^-1^ was applied in three equal splits as BSN, TLN, and PIN, respectively.

The germinated seeds were sown on 12 April in 2019 and 10 April in 2020. The sowing rate was 60 kg ha^-1^ for HHZ and 30 kg ha^-1^ for FLYX1, depending on the difference in tillering ability between inbred and hybrid rice cultivars. Sowing was done at a hill spacing of 10 cm × 25 cm, using a precision rice hill-drop drilling machine (2BDXS-10CP, Shanghai Star CO. Ltd., China). The main crop was harvested by hand on, with a stubble height of 40 cm. Weeds, diseases, and insects were intensively controlled to avoid yield loss.

### 2.3 Sampling and measurements

At the maturity of main crop and ratoon crop, the grain yield was determined from a 5 m^2^ area in the center of each plot. Then the grain were removed by an electric thresher and natural dried until its moisture content can’t change. The grain moisture content was adjusted to the standard moisture content of 0.135 g H_2_O g^-1^ fresh weight by a digital moisture tester (PM-8188, Kitt, Japan). Plants were sampled from an 0.5 m^2^ area in each plot. Panicle number was counted in each sample to determine the panicle number per m^2^, and then plants were separated into straw and panicles. Through hand-threshing, all spikelets parted from the rachis were submerged into the tap water to separate the filled grains and the empty spikelets. Three subsamples with 30 g filled spikelets and three subsamples with 2 g empty spikelets were taken to count the number by grain counting apparatus (SC-G, Wseen, China).

Since nitrogen fertilizer treatment was carried out in the main crop, the following indexes were determined in the main crop to observe the changes of rice plants more directly.

Plants from 0.5 m^2^ area were sampled in each plot at 20 DAS, 52 DAS, panicle initiation, respectively. Then the plant samples were divided into stem and leaves. Dry weight of each organ were measured after oven-drying at 80°C to constant weight and weighed, then grounded into powder for determination of nitrogen content. The nitrogen content was measured using an elemental analyzer (Smart Chem140, Alliance, France). Leaf area index (LAI) of rice was measured by canopy meter (LP-80, Meter, USA) at panicle initiation and heading stage. Two points were measured in each plot, parallel to the row and perpendicular to the row, and the average of the two points was the LAI of the plot. SPAD values of leaves were measured by chlorophyll analyzer (SPAD-502plus, Konica Minolta, Japan) at full heading stage and about 15 days after full heading, and were recorded as SPAD1 and SPAD2, respectively. The senescence rate of leaves is the ratio of the difference between SPAD1 and SPAD2 to the number of recorded days.

At the stage of young ear differentiation, 3 rice plants were selected from each plot and the number of stems (NS) in each plot was recorded. At 6:00 pm, cut off some plants on the ground about 12 cm from each stem, place the pre-weighed (m1) absorbent cotton ball in a ziplock bag and place it at the cutting edge of the stem left in the field, so that the absorbent cotton is in full contact with each stalk, and tie the ziplock bag into the stalk with a rubber band. Retrieve the absorbent cotton bag with wound fluid at 6:00am the next day and weigh it (m2). Root bleeding intensity=(m2-m1)/NS/12. Ten rice seedlings from each plot were separated from the roots and the above-ground parts, and the roots were washed with tap water. Then the root scanning system (WinRHIZO, Regent, Canada) was used to determine the root related apparent properties, including length, surface area, average diameter, number of root tips, number of root crosses, and root dry weight.

### 2.4 Data analysis

Statistical data analysis was performed using analysis of variance (Statistix 9.0, Analytical Software, Tallahassee, FL, USA). Mean comparisons were carried out based on the least significant difference (LSD) test at the 0.05 probability level. All graphical representations of data were performed using SigmaPlot 12.5 (Systat Software Inc., Point Richmond, CA, USA).

## 3 Results

### 3.1 Effective accumulated temperature, effective radiation and rainfall

The effective accumulated temperature, effective radiation and rainfall of rice different growth periods and months were showed in **Figure 1** in 2019 and 2020. The growth period of two varieties is basically the same. As can be seen from **Figure 1**, the total effective accumulated temperature during rice growth in 2019 was 2089.5°C, which was 207.2°C more than the 1882.3°C in 2020. The effective accumulated temperature during in 2019 was slightly lower than 2020, and the effective accumulated temperature in the other two periods was higher than the latter. In particular, the effective accumulated temperature in the HD-MD period in 2019 was 166.7°C higher than that in 2020. The change of two-year effective radiation was similar to the effective accumulated temperature, and the effective radiation in 2019 was 305.1 MJ m^-2^ higher than that in 2020, mainly because the former PI-HD and HD-MD were 208.4 MJ m^-2^ and 210.4 MJ m^-2^ higher than the latter, respectively. The analysis of rainfall showed that the rainfall in the first three periods of the 2020 season was significant higher than that in 2019, and the cumulative rainfall in the former is 291.4 mm higher than that in the latter. The difference was mainly in the PI-HD period, where the rainfall in 2020 was 310.0 mm higher than that in 2019. The higher rainfall in 2020 was the reason for the lower effective accumulated temperature and effective radiation during the PI-HD.

### 3.2 Grain yield and yield components

The grain yield of MC and yield components showed great difference under treatments (**Table 2**). The yield of both varieties treated with N2 was the highest, and the yield was showed as N2>N1>N3>N0, the trend is consistent for two years. The two-year average yield of FLYX1 treated with N2 was 63.6%, 7.6% and 9.4% higher than that of N0, N1 and N3, and that of HHZ treated with N2 was 62.9%, 5.5% and 8.2% higher than that of N0, N1 and N3, respectively. Analysis of yield composition, effective panicles and spikelets number per unit area were the main factors affecting yield. The two-year average panicle of FLYX1 treated with N2 was 62.0%, 2.3% and 9.9% higher than that of N0, N1 and N3, respectively, and that of HHZ treated with N2 was 56.6%, 4.3% and 11.7% higher than that of N0, N1 and N3, respectively. The average number of spikelets per m^2^ of FLYX1 treated with N2 was 67.4%, 7.2% and 13.8% higher than that of N0, N1 and N3, and the average number of spikelets per m^2^ of HHZ treated with N2 was 59.3%, 12.1% and 16.7% higher than that of N0, N1 and N3. The yield of the two varieties in 2020 is lower than that in 2019, which is greatly related to the higher rainfall in 2020, which may directly affect the number of panicles and spikelets.

**Table 2:** The yield and yield components at each treatment during the main crop in 2019 and 2020.

Year	Variety	Treatment	Panicles (No. m^-2^)	Filled grain rate (%)	Spikelets per panicle	Spikelets per m^2^	1000-grain weight (g)	Yield (t ha^-1^)
2019	FLYX1	N0	197.6c	86.2a	165.7b	32755.2d	24.9a	6.8c
		N1	312.7a	81.1d	165.9b	51866.2b	25.5a	10.8b
		N2	316.2a	82.5c	174.1a	55036.1a	25.4a	11.6a
		N3	288.8b	83.9b	166.1b	47987.9c	25.3a	10.6b
	HHZ	N0	232.4d	80.6a	158.3a	36786.6d	21.7a	5.9d
		N1	338.7b	78.1a	156.1a	52867.3b	21.3a	9.0b
		N2	360.5a	78.3a	159.1a	57351.9a	21.6a	9.6a
		N3	318.0c	79.9a	156.3a	49697.6c	21.6a	8.9c
2020	FLYX1	N0	182.0d	90.8a	160.9a	29277.3d	24.5a	6.4c
		N1	288.7b	88.4c	156.3a	45118.7b	24.5a	9.2b
		N2	298.7a	89.8ab	163.6a	48853.2a	24.6a	9.9a
		N3	270.7c	89.1bc	159.8a	43252.1c	24.5a	9.1b
	HHZ	N0	213.3c	90.9a	152.5ab	32526.7c	21.4a	5.7d
		N1	330.0a	89.1a	135.2c	44631.8b	20.9a	8.9b
		N2	337.3a	88.2a	156.8a	52897.6a	21.3a	9.2a
		N3	306.7b	90.5a	145.7b	44674.1b	21.3a	8.6c

Different letters indicate significant difference at 0.05 probability level between different N treatments of the same variety in the same year. N0, no N fertilizer; N1, 180 kg N ha^-1^ was split-applied with 50% as basal nitrogen (BSN) at 1 day before sowing, 20% as tiller nitrogen (TLN) at 20 DAS, and 30% as panicle initiation nitrogen (PIN) at 52 DAS. N2, 180 kg N ha^-1^ was split-applied with 30% as BSN at 1 day before sowing, 40% as TLN at 20 DAS, and 30% as PIN at 52 DAS; N3, 162 kg N ha^-1^ was applied in three equal splits as BSN, TLN, and PIN, respectively.

**Figure 2:**
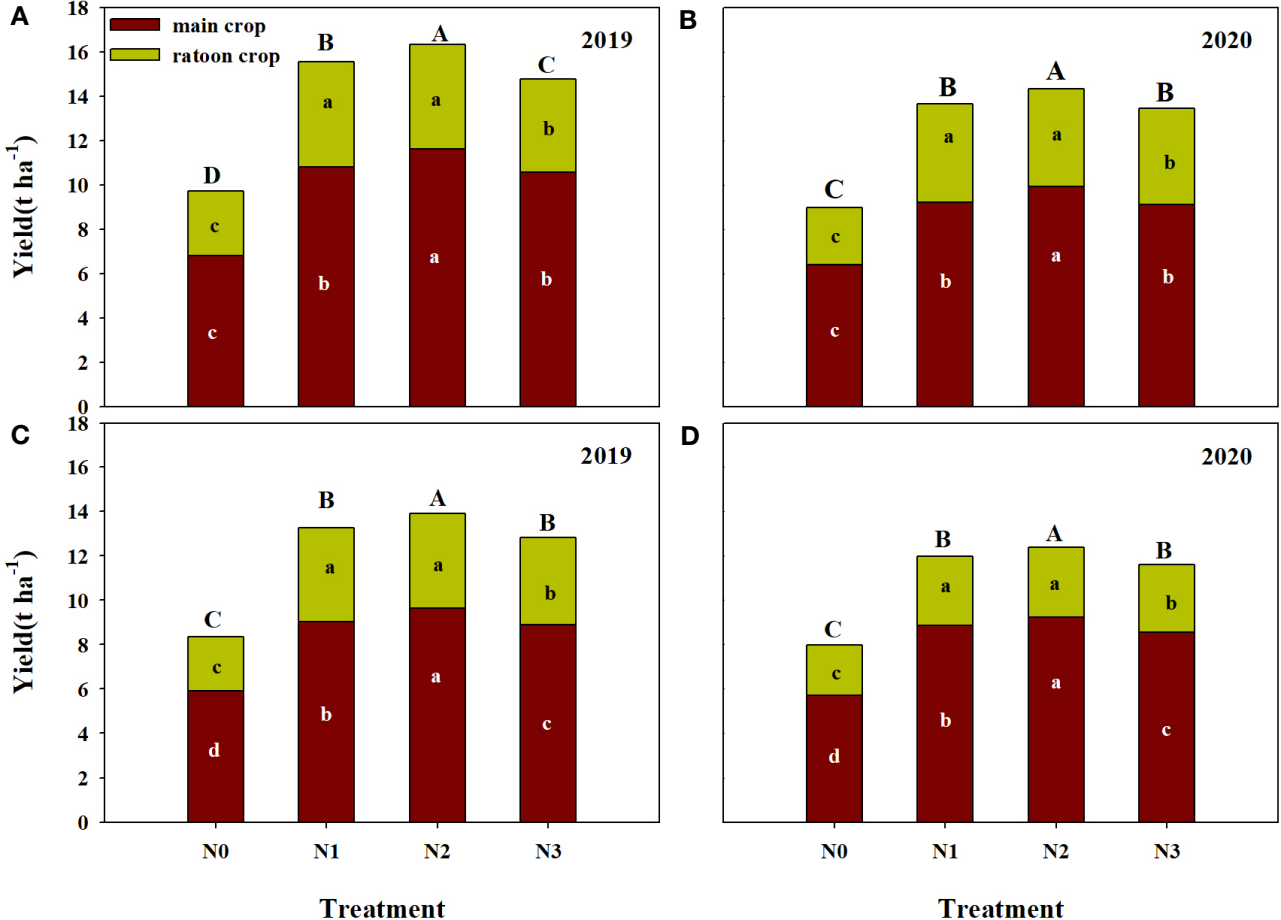
Main crop yield, ratoon crop yield and annual yield compared of FLYX1 and HHZ at each treatment in 2019 and 2020. The lowercase letters on the bar for the same variety and same color in the same year represent significant differences at 0.05 probability level. The capital letters on the bar in the same year represent significant differences at 0.05 probability level. **(A, B)** represent the annual yield of FLYX1 and image **(C, D)** represent the annual yield of HHZ. N0, no N fertilizer; N1, 180 kg N ha^-1^ was split-applied with 50% as basal nitrogen (BSN) at 1 day before sowing, 20% as tiller nitrogen (TLN) at 20 DAS, and 30% as panicle initiation nitrogen (PIN) at 52 DAS. N2, 180 kg N ha^-1^ was split-applied with 30% as BSN at 1 day before sowing, 40% as TLN at 20 DAS, and 30% as PIN at 52 DAS; N3, 162 kg N ha^-1^ was applied in three equal splits as BSN, TLN, and PIN, respectively.

The grain yield of ratoon crop under N1 and N2 treatment showed significant higher than that of N3 and N0 treatment (**Figure 2**). However, the yield of two former treatments was not significant difference. The annual yield of N2 treatment was the highest among all treatments. The two varieties showed the same trend in 2019 and 2020. Except that the annual yield of FLYX1 treated with N1 in 2019 was significantly higher than that of N3 treatment, the annual yield of N1 under other varieties and years was only slightly higher than that of N3 treatment, but the difference between the two was not significant. Two years on average, the annual yield of N2 in FLYX1 were higher than that of N1 and N3 by 4.94% and 8.55%, respectively. And the annual yield of N2 in HHZ were higher than that of N1 and N3 by 4.17% and 7.66%, respectively.

### 3.3 Above ground dry matter mass and total nitrogen accumulation

The comparison of above-ground dry matter mass and nitrogen accumulation of rice plants were indicated significant different at 20 days and 52 days of sowing with each nitrogen fertilizer treatment. As can be seen from **Table 3**, the above-ground dry matter mass and total nitrogen accumulation at 20 days of seeding showed no significant difference between N1, N2 and N3 except that N0 was significant lower than other treatments, and the change trend of the two varieties was consistent, with AB and TN in 2020 slightly lower than that in 2019. Each nitrogen treatment had a significant effect on rice plants 50 DAS. N2 treatment had the highest AB, followed by N1, N3 and N0, and the change trends of the two varieties were consistent, while the AB of the two varieties in 2020 was higher than that in 2019, indicating that the higher accumulated temperature and effective radiation during this period promoted the growth of rice plants (**Figure 1**). The nitrogen accumulation of plants also showed the same trend, N2>N1>N3>N0.

**Table 3:** The above ground dry matter mass(AB) and total nitrogen accumulation(TN) at each treatment during the main crop in 2019 and 2020.

Year	Variety	Treatment	20DFS		52DFS	
			AB (g m^-2^)	TN (g m^-2^)	AB (g m^-2^)	TN (g m^-2^)
2019	FLYX1	N0	2.10b	0.019b	588.0c	6.63c
		N1	7.97a	0.091a	810.0ab	8.19a
		N2	7.90a	0.091a	840.4a	8.43a
		N3	7.83a	0.090a	761.4b	7.66b
	HHZ	N0	1.50b	0.014b	411.5c	3.12c
		N1	5.80a	0.068a	633.4ab	6.75b
		N2	5.73a	0.069a	661.5a	7.67a
		N3	5.63a	0.066a	602.7b	6.26b
2020	FLYX1	N0	1.90b	0.014b	372.2c	4.86c
		N1	7.70a	0.084a	988.5a	9.52a
		N2	7.63a	0.084a	999.1a	9.83a
		N3	7.53a	0.083a	874.5b	8.61b
	HHZ	N0	1.30b	0.010b	375.2c	3.13c
		N1	5.40a	0.058ab	881.3a	8.43a
		N2	5.30a	0.060a	900.2a	8.71a
		N3	5.27a	0.060a	826.0b	7.67b

Different letters indicate significant difference at 0.05 probability level between different N treatments of the same variety in the same year. N0, no N fertilizer; N1, 180 kg N ha^-1^ was split-applied with 50% as basal nitrogen (BSN) at 1 day before sowing, 20% as tiller nitrogen (TLN) at 20 DAS, and 30% as panicle initiation nitrogen (PIN) at 52 DAS. N2, 180 kg N ha^-1^ was split-applied with 30% as BSN at 1 day before sowing, 40% as TLN at 20 DAS, and 30% as PIN at 52 DAS; N3, 162 kg N ha^-1^ was applied in three equal splits as BSN, TLN, and PIN, respectively.

### 3.4 LAI value in PI and HD

**Figure 3:**
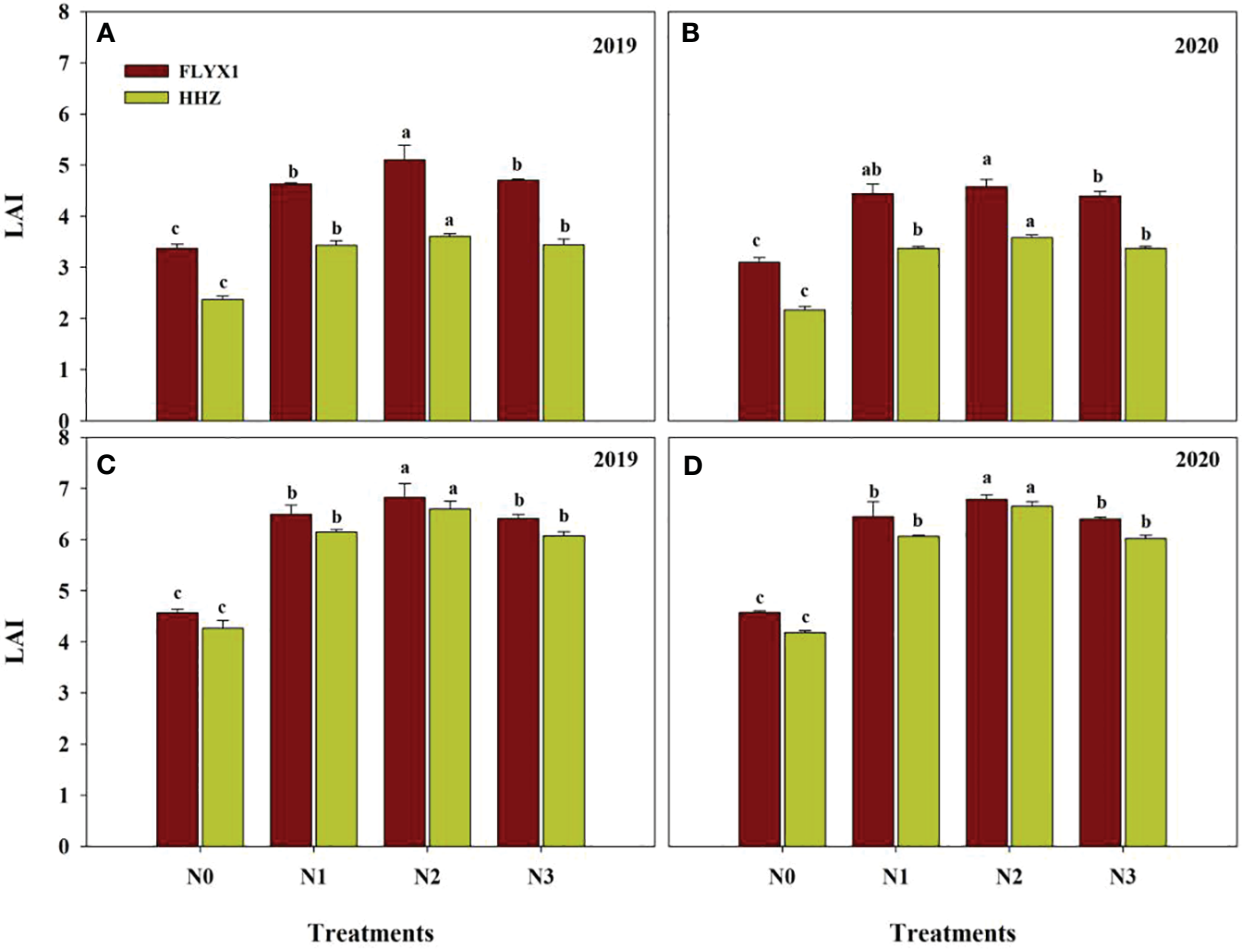
LAI compared of FLYX1 and HHZ at each treatment during the main crop in 2019 and 2020. The lowercase letters on the bar figure for the same variety in the same year represent significant differences at 0.05 probability level. **(A, B)** represent LAI at PI and **(C, D)** represent LAI at HD. N0, no N fertilizer; N1, 180 kg N ha^-1^ was split-applied with 50% as basal nitrogen (BSN) at 1 day before sowing, 20% as tiller nitrogen (TLN) at 20 DAS, and 30% as panicle initiation nitrogen (PIN) at 52 DAS. N2, 180 kg N ha^-1^ was split-applied with 30% as BSN at 1 day before sowing, 40% as TLN at 20 DAS, and 30% as PIN at 52 DAS; N3, 162 kg N ha^-1^ was applied in three equal splits as BSN, TLN, and PIN, respectively.

All treatments had significant effects on LAI at PI stage and HD stage of the two varieties and two years (**Figure 3**). As can be seen from the **Figure 3**, LAI under N2 treatment was the highest in PI stage, while there was no significant difference between LAI under N1 and N3 treatments, and LAI under all three treatments was significant higher than that under N0. At the same time, the average LAI of FLYX1 treatment was 35.3% higher than that of HHZ, indicating that the former group was constructed faster in the early stage. In HD phase, LAI under N2 treatment was also significant higher than that under other treatments, while there was no significant difference between LAI under N1 and N3 treatment, and LAI of the three treatments was significant higher than N0. Under the same treatment, the influence of different varieties on LAI was reduced. Across treatments and years, the average LAI of FLYX1 was only 5.5% higher than HHZ at HD stage.

### 3.5 Leaf senescence rate

**Figure 4:**
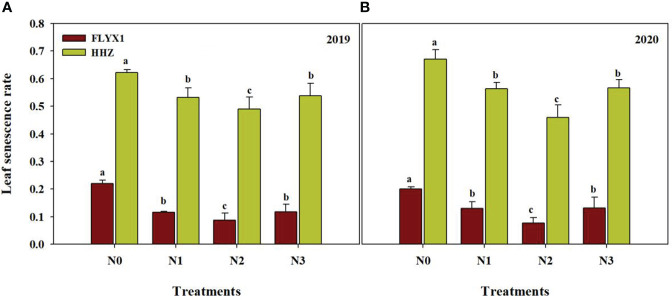
Leaf senescence rate of FLYX1 and HHZ at each treatment during the main crop in 2019 **(A)** and 2020 **(B)**. The different letters between different treatments in the same year all showed significant differences at 0.05 probability level. N0, no N fertilizer; N1, 180 kg N ha^-1^ was split-applied with 50% as basal nitrogen (BSN) at 1 day before sowing, 20% as tiller nitrogen (TLN) at 20 DAS, and 30% as panicle initiation nitrogen (PIN) at 52 DAS. N2, 180 kg N ha^-1^ was split-applied with 30% as BSN at 1 day before sowing, 40% as TLN at 20 DAS, and 30% as PIN at 52 DAS; N3, 162 kg N ha^-1^ was applied in three equal splits as BSN, TLN, and PIN, respectively.

The leaf senescence rate of the two varieties showed significant difference under each treatment. As can be seen from the **Figure 4**, there were significant differences in leaf senescence rate under different nitrogen treatments, and the leaf senescence rate under N0 treatment was the highest, which was significant higher than other treatments, and the change trend of the two varieties was consistent in two years. The leaf senescence rate of FLYX1 was 60.8%, 32.8% and 33.9% lower than that of N0, N1 and N3, and that of HHZ was 26.6%, 13.4% and 14.2% lower than that of N0, N1 and N3, respectively. At the same time, the leaf senescence rate of FLYX1 was significant lower than that of HHZ, and the average value of the former under each treatment was 75.8% lower than that of the latter, indicating that the ability of FLYX1 leaves to maintain higher chlorophyll content in the later stage was stronger than that of HHZ.

### 3.6 Root growth status

The root system is the organ most directly affected by soil nutrients. In this study, different nitrogen fertilizer treatments had significant effects on root growth status. From **Table 4**, the root length, surface area, root diameter, number of root tips, forks number and dry matter weight under N2 treatment were higher than those under other treatments, and the performance of N3, N1 and N0 decreased successively, and the change trend of the two varieties was consistent in two years. At the same time, the root various measurement indicators of FLYX1 treatment was higher than the HHZ of the same treatment, and the root performance of the same treatment in 2020 was lower than that in 2019.

**Table 4:** Root phenotype analysis at each treatment during the main crop in 2019 and 2020.

Year	Variety	Treatment	Length (cm)	SA (cm^2^)	AD (mm)	Tips	Forks	Weight (g)
2019	FLYX1	N0	920.5c	81.5b	0.90b	5062.6c	5564c	0.121b
		N1	936.9bc	89.6b	0.92b	5577.7b	6579b	0.140b
		N2	1091.7a	123.8a	1.36a	6533.3a	7629a	0.183a
		N3	966.1ab	110.8a	1.21ab	5570.7b	6892b	0.150ab
	HHZ	N0	894.6c	99.2b	0.88b	4133.1c	5015d	0.091b
		N1	911.3bc	111.6a	1.09ab	4668.3b	5427c	0.120b
		N2	957.0a	118.2a	1.17a	5683.3a	6955a	0.163a
		N3	924.6b	112.9a	1.14ab	4703.7b	5966b	0.117b
2020	FLYX1	N0	918.8c	80.2c	0.85b	3889.1c	5455c	0.115b
		N1	921.7bc	85.5c	0.87b	4185.3b	6288b	0.120b
		N2	1011.8a	120.2a	1.22a	5582.0a	7377a	0.157a
		N3	952.5b	108.5b	1.15a	4357.3b	6771b	0.123b
	HHZ	N0	700.1c	72.5c	0.80b	3128.5c	5135c	0.089b
		N1	714.8c	84.4b	0.82b	3742.7b	5557b	0.110b
		N2	772.2a	103.3a	1.07a	4024.7a	6493a	0.157a
		N3	749.7b	88.2b	0.97a	3753.3b	5354bc	0.117b

Different letters indicate significant difference at 0.05 probability level between different N treatments of the same variety in the same year. SA, surface area; AD, average diameter, Tips, number of root tips; Forks, number of root crosses; Weight, root dry weight. N0, no N fertilizer; N1, 180 kg N ha^-1^ was split-applied with 50% as basal nitrogen (BSN) at 1 day before sowing, 20% as tiller nitrogen (TLN) at 20 DAS, and 30% as panicle initiation nitrogen (PIN) at 52 DAS. N2, 180 kg N ha^-1^ was split-applied with 30% as BSN at 1 day before sowing, 40% as TLN at 20 DAS, and 30% as PIN at 52 DAS; N3, 162 kg N ha^-1^ was applied in three equal splits as BSN, TLN, and PIN, respectively.

### 3.7 Root bleeding intensity

**Figure 5:**
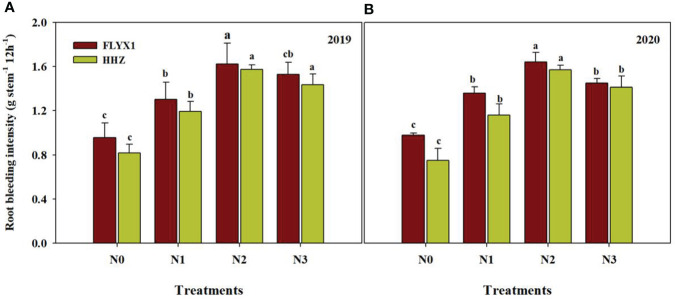
Root bleeding intensity of FLYX1 and HHZ at each treatment during the main crop in 2019 **(A)** and 2020 **(B)**. The different letters between different treatments in the same year all showed significant differences at 0.05 probability level. N0, no N fertilizer; N1, 180 kg N ha^-1^ was split-applied with 50% as basal nitrogen (BSN) at 1 day before sowing, 20% as tiller nitrogen (TLN) at 20 DAS, and 30% as panicle initiation nitrogen (PIN) at 52 DAS. N2, 180 kg N ha^-1^ was split-applied with 30% as BSN at 1 day before sowing, 40% as TLN at 20 DAS, and 30% as PIN at 52 DAS; N3, 162 kg N ha^-1^ was applied in three equal splits as BSN, TLN, and PIN, respectively.

The amount of crop root bleeding intensity indicates the health state of the plant. In this study, different nitrogen fertilizer treatments had significant effects on the root activity of the two varieties (**Figure 5**). It can be seen that the root injury flow rate under N2 treatment was significant higher than that under other treatments, that under FLIYX1 treatment was 69.1%, 22.6% and 9.8% higher than that under N0/N1 and N3, and that under HHZ treatment was 101.7%, 33.7% and 10.7% higher, respectively. At the same time, the root bleeding intensity of FLYX1 under the same treatment was higher than HHZ.

**Figure 6:**
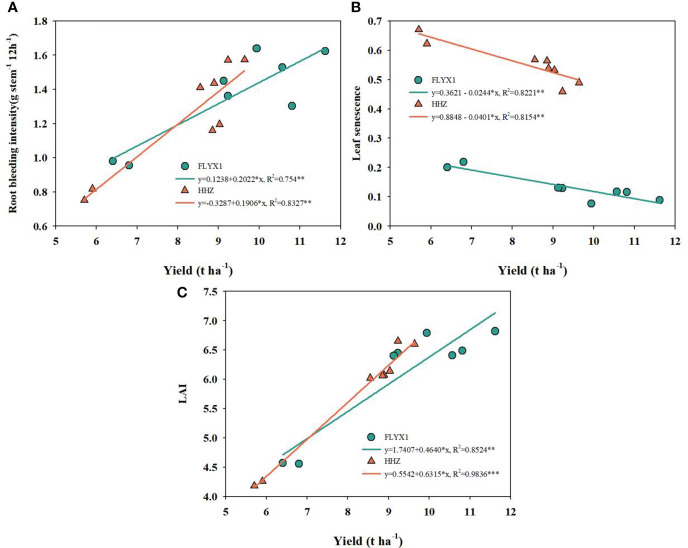
The correlation of yield with root bleeding intensity, leaf senescence and LAI in 2019 and 2020. * and ** represent significant at the 0.05 probability level. **(A–C)** show the correlation between yield and root bleeding intensity, leaf senescence and LAI, respectively.

**Figure 6** shows the correlation analysis of root wound flow, leaf senescence and leaf area index with yield. As can be seen from the figure, the three indexes are highly significant correlated with yield. The root wound flow rate of FLYX1 and HHZ is significant positively correlated with yield, and the correlation coefficients R^2^ reach 0.754 and 0.833, respectively. The leaf aging rate of FLYX1 and HHZ is significant negatively correlated with yield, and the correlation coefficients R^2^ reach 0.822 and 0.815, respectively. In addition, the leaf area index of the two groups was also significant positively correlated with yield, and the correlation coefficients R^2^ reached 0.852 and 0.984, respectively.

## 4 Discussion

In recent years, key measures such as reasonable sowing date, fertilizer and water management and stubble height have significant promoted the increase of grain yield of ratoon rice in China (Peng et al., 2023), among which the scale of production of ratoon rice has been increasing and has attracted more and more attention (Wang et al., 2017). Compared with traditional transplanting, rice direct seeding technology can save fresh water better, reduce labor force and improve production efficiency (Tao et al., 2016). The DSRR mode in this study is a planting mode that combines the advantages of direct seeding rice and ratoon rice (Dong et al., 2017). Compared with transplanting ratoon rice, its nutrient management method is not suitable for direct regenerated rice, because the field environment after sowing is very different between the two plant modes. In this study, the rice yield under the treatment of reducing the amount of base fertilizer and increasing the amount of tillering fertilizer (N2) was significant 6.6% higher than that under the traditional management (N1), and 63.3% higher than that of N0 (**Table 2**). This is similar to the results of previous studies that significant increased the yield of direct seeding rice by means of front-to-back nitrogen transfer (Duan et al., 2020; Jiang et al., 2021). In this study, the yield of N3 treatment showed a tendency to decrease compared with N1 treatment, while N2 treatment was 8.8% higher than N3 treatment (**Table 2**), indicating that for DSRR, the application of part of nitrogen fertilizer could be delayed, while direct reduction was not conducive to yield improvement. According to Dong (Dong et al., 2017), higher yields can be achieved if one third of the total nitrogen fertilizer was applied to base fertilizer and all other nitrogen fertilizers are applied at a later stage. The main reason of yield difference of this study were the difference in effective panicle and spikelet number per unit area. The effective panicle of N2 treatment was 59.3%, 3.3% and 10.8% higher than that of N0/N1/N3, and the spikelet number per unit area was 63.3%, 9.6% and 15.4% higher, respectively (**Table 2**). The results showed that the difference of nitrogen fertilizer treatment had great influence on the plant population in the early stage. Increasing the amount of fertilizer at seedling stage significant promoted tillering, and the maximum number of tillers was significant positively correlated with the amount of fertilizer at seedling stage (Tan et al., 2016). However, the amount of base fertilizer in N1 treatment was large, and the plant utilization was not much, resulting in partial nitrogen loss and the total amount of fertilizer acting on tillering part is reduced. As rainfall increases, fertilizer loss to the environment will also increase (Chen et al., 2017), because nitrogen concentration in surface water will remain high for 7-10 days after fertilization (Davis et al., 2016). Reasonable reduction of basal fertilizer application may be an effective way to improve nitrogen use efficiency, decrease the loss of nitrogen to environment and increase the planting income in DSRR mode. From **Figure 2**, the annual yield of N1 was highest value among all the treatments, that significant improved the nitrogen use efficiency. At the same time, the reduction of total nitrogen application further reduced the risk of nitrogen loss, and was beneficial to reduce production input and increase production income (Dong et al., 2017).

Dry matter accumulation of rice population is a direct reflection of soil nutrient supply (Zhang et al., 2011). The results of this study showed that the above-ground dry matter quality at 20 days after seeding was significant lower than that of N0, and the other three treatments showed no significant difference under the same variety, while the above-ground dry matter quality at 50 days after seeding gradually showed differences under different treatments (**Table 3**). Just as the results of Tian et al.’s study showed that because there was no seedling cultivation link, the vegetative growth period of direct seeding rice was shortened, with relatively low substance accumulation in the early stage, large growth in the middle and late stage, and fast population development (Tian et al., 2016). In this study, after seeding in DSRR, the temperature was not high and unstable (**Figure 1**), and the plant production process such as emergence and tillering was slower than that of direct seeding in medium rice. When the temperature is stable above 15°C, it is conducive to dry matter accumulation (Wang et al., 2013), and then the demand for nutrients will be more. In this study, the first topdressing time was 20 days after seeding, and N2 treatment just increased the amount of nitrogen fertilizer applied, making LAI significantly higher than other treatments in PI and HD periods. The senescence process of leaves in the later stage is also a reflection of changes in soil nutrient supply and transfer (Lee and Masclaux-Daubresse, 2021). The leaf senescence rate of N2 treatment was significant lower than that of other treatments, followed by N1 and N3 treatments, and N0 treatment had the fastest senescence rate, and the change trend of the two varieties was consistent in two years (**Figure 4**), indicating that the traditional fertilization mode (N1) under DSRR could easily cause premature leaf senescence in the later stage, which was not conducive to the light energy conversion and transport in the later stage. As Liu (Liu et al., 2019) showed, postponing the application of early fertilizer to the period between tillering and young panicle initiation had positive effects on maintaining late leaf color, higher photosynthetic accumulation in rice canopy and grain setting rate. In addition, in this study, the late senescence rate of leaves of HHZ was significant higher than that of FLYX1, indicating that the performance of hybrid rice and inbred rice in DSRR was significant different, and a better plant state of hybrid rice was conducive to reducing production inputs such as fertilizer (Yuan et al., 2017).

The root system is an effective reflection of the growth state of direct-seeding rice (Islam et al., 2023), and different nitrogen fertilizer application rates significantly affect the root development length and root activity of rice (Xu et al., 2019). In this study, the root length, surface area, diameter and dry matter mass of N2 treatment were significant higher than those of N1 and N0 treatment (**Table 4**). Combined with the above analysis of dry matter accumulation (**Table 3**), it was indicated that DSRR mode could effectively promote the growth of aboveground plant and roots under N2 treatment, while the promotion effect of lower nitrogen fertilizer was lower. Studies have shown that increasing seeding density can significantly improve root morphology and distribution under low nitrogen fertilizer application, which has a positive effect on direct seeding rice yield (Deng et al., 2020). The DSRR mode may also be able to further improve the absorption and utilization of nitrogen fertilizer by increasing the sowing amount. As can be seen from **Figure 5**, the single stem bleeding intensity of N3 treatment is higher than that of N1 treatment, and the effective panicle number of the former is lower than that of N1 treatment, indicating that increasing the number of single stems may improve the absorption and utilization of fertilizer, but cannot improve the root vitality of a single rice stem. It also has a great impact on the lodging resistance of roots (Zhang et al., 2021). **Figure 6** shows that there is a significant positive correlation between root activity and yield, which further indicates the importance of root activity for yield. Studies have been conducted to improve the yield of direct seeding rice by improving the comprehensive phenotype of roots (Ajmera et al., 2022), which is an approach for rice breeding and may also provide an important indicator for evaluating the high-efficiency production of DSRR system.

## 5 Conclusions

It was a useful method that transferring 20% of total nitrogen from basal fertilizer to tillering stage in DSRR can effectively promote the accumulation of dry matter in the above and underground parts, maintain high root vitality, promote population construction, postpone senescence process of leaves, promote the accumulation, and significant increase the main crop yield and the annual yield. On the basis of the above method, further reducing the amount of tillering fertilizer by 10% will have a negative impact on the yield, but the yield will not be reduced compared with the traditional fertilization method, which is beneficial to improve the production efficiency of nitrogen fertilizer and improve the planting income in DSRR system.

## Data availability statement

The original contributions presented in the study are included in the article/supplementary material. Further inquiries can be directed to the corresponding author.

## Author contributions

YL: Data curation, Investigation, Methodology, Writing – original draft, Writing – review & editing, Formal Analysis, Resources. ZLZ: Data curation, Investigation, Methodology, Writing – original draft, Writing – review & editing, Formal Analysis, Resources. BW: Investigation, Resources, Writing – review & editing. ZSZ: Investigation, Resources, Writing – review & editing. YYL: Investigation, Writing – original draft. JC: Data curation, Funding acquisition, Project administration, Supervision, Writing – review & editing.
